# Associations between Ultrasound Measures of Abdominal Fat Distribution and Indices of Glucose Metabolism in a Population at High Risk of Type 2 Diabetes: The ADDITION-PRO Study

**DOI:** 10.1371/journal.pone.0123062

**Published:** 2015-04-07

**Authors:** Annelotte Philipsen, Marit E. Jørgensen, Dorte Vistisen, Annelli Sandbaek, Thomas P. Almdal, Jens S. Christiansen, Torsten Lauritzen, Daniel R. Witte

**Affiliations:** 1 Steno Diabetes Center A/S, Gentofte, Denmark; 2 Department of Public Health, Section for General Practice, Aarhus University, Aarhus, Denmark; 3 Department of Internal Medicine F, Gentofte Hospital, Gentofte, Denmark; 4 Department of Endocrinology, Aarhus University Hospital, Aarhus, Denmark; University of Florida, UNITED STATES

## Abstract

**Aims:**

Visceral adipose tissue measured by CT or MRI is strongly associated with an adverse metabolic risk profile. We assessed whether similar associations can be found with ultrasonography, by quantifying the strength of the relationship between different measures of obesity and indices of glucose metabolism in a population at high risk of type 2 diabetes.

**Methods:**

A cross-sectional analysis of 1342 participants of the ADDITION-PRO study. We measured visceral adipose tissue and subcutaneous adipose tissue with ultrasonography, anthropometrics and body fat percentage by bioelectrical impedance. Indices of glucose metabolism were derived from a three point oral glucose tolerance test. Linear regression of obesity measures on indices of glucose metabolism was performed.

**Results:**

Mean age was 66.2 years, BMI 26.9kg/m^2^, subcutaneous adipose tissue 2.5cm and visceral adipose tissue 8.0cm. All measures of obesity were positively associated with indicators of glycaemia and inversely associated with indicators of insulin sensitivity. Associations were of equivalent magnitude except for subcutaneous adipose tissue and the visceral/subcutaneous adipose tissue ratio, which showed weaker associations. One standard deviation difference in BMI, visceral adipose tissue, waist circumference, waist/height ratio and body fat percentage corresponded approximately to 0.2mmol/l higher fasting glucose, 0.7mmol/l higher 2-hr glucose, 0.06-0.1% higher HbA1c, 30 % lower HOMA index of insulin sensitivity, 20% lower Gutt’s index of insulin sensitivity, and 100 unit higher Stumvoll’s index of beta-cell function. After adjustment for waist circumference visceral adipose tissue was still significantly associated with glucose intolerance and insulin resistance, whereas there was a trend towards inverse or no associations with subcutaneous adipose tissue. After adjustment, a 1cm increase in visceral adipose tissue was associated with ~5% lower insulin sensitivity (p≤0.0004) and ~0.18mmol/l higher 2-hr glucose (p≤0.001).

**Conclusion:**

Visceral and subcutaneous adipose tissue assessed by ultrasonography are significantly associated with glucose metabolism, even after adjustment for other measures of obesity.

## Introduction

Obesity is a major risk factor for the development of type 2 diabetes (DM), but the risk is heterogeneous among obese individuals [1]. Independent of overall obesity, central obesity is an established risk factor for the disease [2]. Evidence has emerged, however, that particularly patterns of visceral rather than subcutaneous fat distribution around the waist may confer increased metabolic risk. In recent years studies spanning populations of different genders, ages, BMI levels and ethnicities, have indicated that visceral adipose tissue (VAT) plays a different and more adverse metabolic role than subcutaneous adipose tissue (SAT) [3–7]. VAT is thought to be an indicator of the relative inability of SAT to store more energy during continued positive caloric balance. Thus people who are not able to store their energy surplus in SAT will be characterised by accumulation of fat at undesired sites such as intra-abdominally [8].

The distribution of abdominal fat as visceral and subcutaneous fat is a dimension of obesity that is not directly captured by commonly used anthropometric measures such as body mass index (BMI) or waist circumference. CT or MRI imaging techniques are considered gold standard for assessing abdominal fat distribution but are typically not feasible in large scale studies or in clinical practice due to costs, including the need for heavily used clinical equipment, and in the case of CT also due to radiation exposure. Cheaper and more accessible non-invasive ultrasonography has been validated against CT and MRI as a method of assessing abdominal fat distribution [9–12], and the method is now in use [13–16].

The purpose of this study was to examine if abdominal fat distribution, when assessed by ultrasound, is associated with detailed indices of glucose metabolism derived from a 3 point OGTT, and whether this assessment adds information regarding such risk above that obtained from other commonly used measures of obesity.

## Materials and Methods

### Study population

We investigated participants who attended the 2009–2011 follow up health examination of the ADDITION-PRO study, a longitudinal cohort of participants at high risk of developing type 2 DM identified through stepwise screening in Danish general practice (2001–2006), and performed a cross-sectional analysis. The details of this study including the screening procedure have been described elsewhere [17]. In brief, 2082 people representing specific baseline diabetes risk groups, took part in the 2009–2011 ADDITION-PRO examination. These groups were: combined impaired fasting glycaemia and impaired glucose tolerance, isolated impaired fasting glycaemia, isolated impaired glucose tolerance, and high risk based on questionnaire data but normal glucose tolerance. The cohort also included a small group who were at low risk with normal glucose tolerance. The health assessment involved detailed measurement of anthropometry, biochemistry and physical activity, and completion of validated questionnaires. Data were collected at four study centres in Denmark. This study was a subgroup analysis of participants from two of the study centres where ultrasound assessments of abdominal fat distribution were performed.

### Obesity measures

Waist circumference was measured to the nearest 0.1cm as the midpoint between the iliac crest and the lower rib with the participant standing using a D-loop tape. Height was measured with the participants wearing light indoor clothing but no shoes to the nearest 0.1cm with a stadiometer (Seca, Medical Scales and Measuring Systems, Hamburg, Germany). Body fat percentage and weight were assessed by bioelectrical impedance using a leg-to-leg Tanita Body Composition Analyser (Tokyo, Japan). BMI was defined as weight (kg) divided by height (m) squared. Waist-to-height ratio was defined as waist circumference (cm) divided by height (cm).

**Fig 1:**
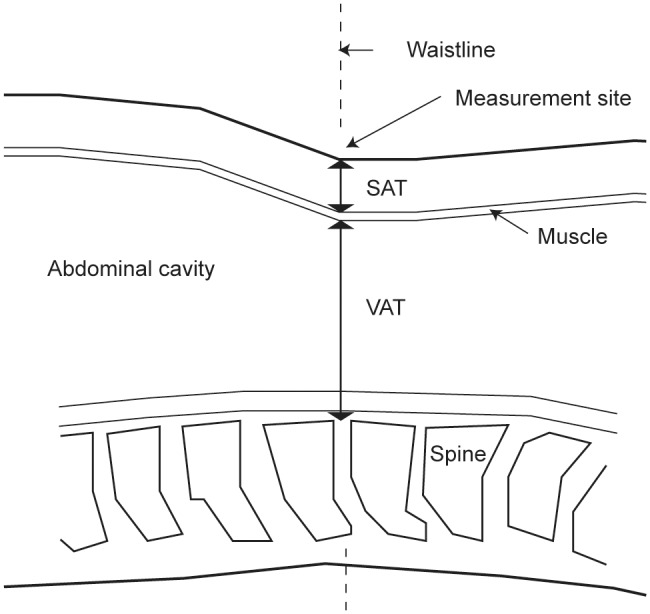
Measurement of visceral adipose tissue (VAT) and subcutaneous adipose tissue (SAT) with ultrasonography. Both measurements were performed where the xiphoid line crosses the waist line.

**Fig 2:**
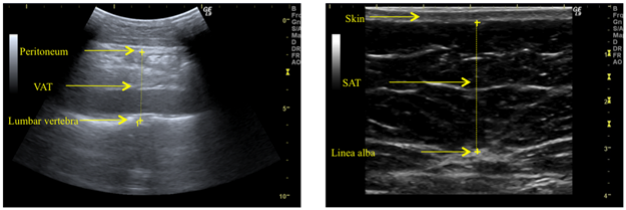
Examples of ultrasound images of VAT and SAT.

Abdominal fat distribution was assessed by ultrasonography (Logiq 9 machine, GE Healthcare, Waukesha, WI, USA) according to a strict validated protocol [9–11]. With the participant lying down, VAT thickness was defined as the depth (cm) from the peritoneum to the lumbar spine, and SAT thickness as the depth (cm) from the skin to the linea alba. Both measurements were made where the xyphoid line crosses the waistline (Fig 1). Visceral fat was measured using a 4C abdominal convex transducer placed longitudinally and subcutaneous fat with a 9L small parts linear transducer placed transversely. Scan depth was individually set for each image in order to best visualise anatomical structures. Measurements were performed at the end of a quiet expiration using minimal pressure on the transducer so as not to compress the fat tissue. Fig 2 shows examples of ultrasound assessment of VAT and SAT.

### Indices of glucose metabolism

Participants underwent a 75g oral glucose tolerance test (OGTT) after an overnight fast of at least 8 hours. Venous blood samples were drawn for the measurement of plasma glucose and serum insulin at times 0, 30 and 120 minutes. From the fasting sample HbA_1c_ was also measured. Serum insulin was measured by immunoassay (AutoDELFIA, Perkin Elmer, Massachusetts, USA). HbA_1c_ was measured by ion-exchange high-performance liquid chromatography (TOSOH G7, Tokyo, Japan). Between 2009 and 2010 plasma glucose was measured using the Hitachi 912 system (Roche Diagnostics, Mannheim Germany). In 2010 the study laboratory went over to using the Vitros 5600 Integrated System (Ortho Clinical Diagnostics, Illkirch Cedex, France) instead. All glucose values measured on the Vitros system were therefore converted to correspond to Hitachi values using regression equations from validation analysis done by the study laboratory [17].

The OGTT was used to derive indices of glucose metabolism. HOMA index of insulin sensitivity was derived from homeostasis model assessment according to the approximation formulae [18]:

$$HOMA-S\;=\;\frac{\;22.5}{\;({glucose}_{t0\;}\times (\frac{{insulin}_{t0}}{6.945}))}$$

(units (mmol/l×mU/l)^-1^).

To obtain an estimate of insulin sensitivity in peripheral tissue, Gutt’s index of insulin sensitivity was calculated as [19]:

$${ISI}_{0,120}\;=\;\frac{75000+180\left({glucose}_{t0\;}-\;{glucose}_{t120\;}\right)\times 0.19\;\times body\;weight}{120\times \left(\frac{{glucose}_{t0\;}+\;{glucose}_{t120\;}}{2}\right)\times \log((({insulin}_{t0}+{insulin}_{t120\;})/6.945)/2)\;}$$

(units mg×$l^{2}$/mmol×mU×min).

As a measure of beta cell function Stumvoll’s index of early phase insulin release was calculated as [20,21]:

$$Stumvoll\;=\;1.238+1.829\times {insulin}_{t30}-138.7\times {glucose}_{t30}+3.772\times {insulin}_{t0}$$

(units for p-glucose mmol/l and p-insulin pmol/l).

Thirty participants had negative values for this index. These values were changed to an arbitrary value close to zero (0.01) in the subsequent analysis.

### Covariate measurements

Physical activity was assessed using a modified Danish version of the validated “recent physical activity questionnaire” (RPAQ) [22]. It contains questions on type, frequency, intensity and context of physical activity during the four weeks prior to filling out the questionnaire. From information in this questionnaire the physical activity energy expenditure (PAEE) was calculated for each participant using the 2005 Oxford model [23]. From a general questionnaire, self-reported information on current medication and smoking status was obtained. Current hormone replacement therapy was defined as use of any medication with an ATC code belonging to the G03 group of the World Health Organisation’s classification system. The unique Danish civil registration number provided information on age and sex.

### Ethical considerations

The ADDITION-PRO study was approved by the scientific ethics committee of Central Denmark Region (approval number M-20080229) and performed in accordance with the Helsinki declaration. All participants gave written informed consent.

### Statistical analysis

Linear regression analysis was performed to assess the association between measures of obesity as explanatory variables and indices of glucose metabolism as outcome variables. Measures of obesity were standardized (sex-specific) in order to facilitate comparisons of the strengths of the associations. Skewed outcome measures (all three insulin measurements, HOMA insulin sensitivity and Gutt’s insulin sensitivity index) were log-transformed to improve normality prior to analysis.

In a first analysis, adjustment was made for age and sex. Next, adjustment was also made for current smoking, current hormone replacement therapy and physical activity. In all analysis, a cross-product term between obesity measures and sex was included. The associations were reported for men and women separately, and differences between the sexes were tested using t-tests. Complete case analysis defined the subset of data used.

Analysis were repeated for non-standardized VAT, SAT and fat percentage to allow clinical interpretation of the results in otherwise identical models. Furthermore, this analysis was repeated with further adjustment for waist circumference and body fat percentage. This allowed quantification of the added value of measuring VAT and SAT by ultrasound.

All analyses were performed using the statistical software SAS version 9.2 (SAS Institute Inc., Cary, NC, USA.). Forest plots were made using the statistical software R version 9.15.2 [24].

## Results

The dataset for this study is available in supporting information file S1 Dataset.

### Study Population

**Fig 3:**
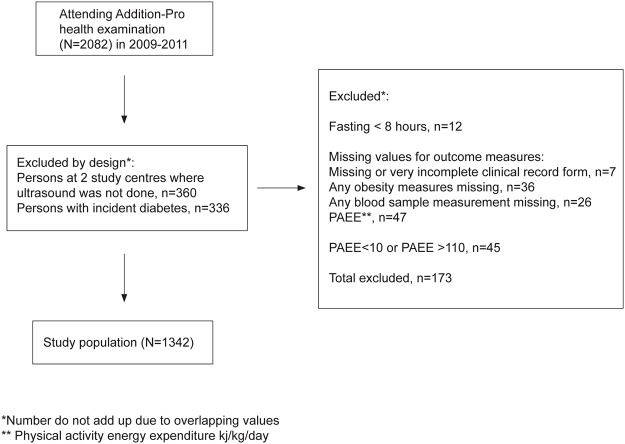
Study population.

In the present study, we excluded participants with incident diabetes since screening as an OGTT had not been performed on these participants (n = 336). 704 men and 638 women were available for complete case analysis; see Fig 3 for detail on the reasons for exclusion of participants to obtain the study sample. At baseline screening our participants had been distributed among diabetes risk groups as follows: combined impaired fasting glycaemia and impaired glucose tolerance (n = 31), isolated impaired fasting glycaemia (n = 109), isolated impaired glucose tolerance (n = 102), high risk based on questionnaire data but normal glucose tolerance (n = 1032), and low risk with normal glucose tolerance (n = 168). The mean age of participants was 66.2 years (SD 7.0). Values for BMI were concentrated in the normal to overweight range. Almost all participants were Caucasian. Table 1 summarises selected study sample characteristics.

**Table 1:** Characteristics of the study sample.

	Men	Women
N	704	638
Age	66.7 (62.7–71.8)	66.1 (60.8–71.4)
Ethnicity, Caucasian N (%)	691 (98%)	616 (97%)
BMI (kg/m2)	26.8 (24.8–29.6)	25.7 (22.7–28.9)
Waist circumference (cm)	99.5 (92.1–107.3)	87.7 (80.0–96.7)
Waist-to-height ratio (cm/cm)	0.56 (0.52–0.61)	0.54 (0.48–0.59)
Visceral fat (cm)	8.7 (7.1–10.5)	6.5 (5.3–8.0)
Subcutaneous fat (cm)	2.2 (1.6–2.8)	2.8 (2.1–3.5)
Visceral/subcutaneous fat ratio	4.0 (2.9–5.7)	2.4 (1.9–3.3)
Body fat percentage (%)	26.6 (22.4–30.4)	37.5 (32.7–42.2)
P-glucose, fasting (mmol/L)	6.0 (5.6–6.5)	5.8 (5.5–6.2)
P-glucose, t = 30 min (mmol/L)	9.2 (8.3–10.2)	8.7 (7.7–9.8)
P-glucose, t = 120 min (mmol/L)	6.4 (5.3–7.9)	6.2 (5.1–7.3)
S-insulin, fasting (pmol/L)	38 (25–60)	35 (24–49)
S-insulin, t = 30 min (pmol/L)	220 (147–324)	215 (151–303)
S-insulin, t = 120min (pmol/L)	182 (105–326)	184 (115–280)
HbA1c (%)	5.6 (5.4–5.9)	5.7 (5.5–5.9)
HbA1c (mmol/mol)	38 (36–41.0)	39 (37–41.0)
Current smoker N (%)	516 (73)	341 (53)
Hormone replacement therapy N (%)	0	59 (9)
Physical activity energy expenditure	46.2 (34.3–58.3)	48.0 (37.1–60.9)
(kj kg-1 day-1)		

Data are median (IQR) or percentage. N, number of observations.

### Associations of obesity measures with indices of glucose metabolism

**Fig 4:**
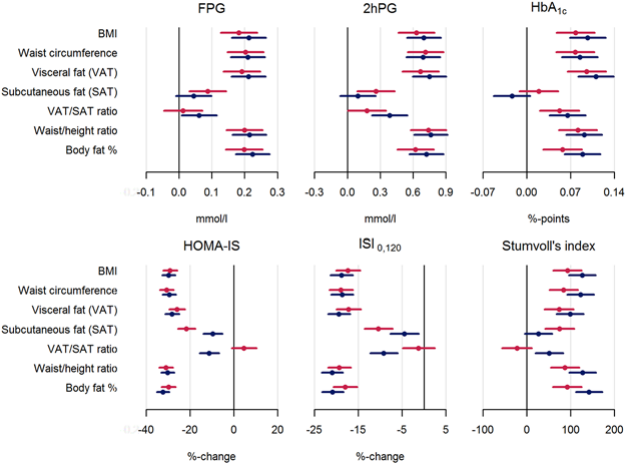
Linear associations between standardized obesity measures and indices of glucose metabolism (women (red); men (blue)): adjusted for age, HRT, smoking status, and physical activity.

Forest plots (Fig 4) illustrate results from linear regression models of standardized obesity measures on indices of glucose metabolism adjusted for all confounders. Results obtained from a model with adjustment for only age and sex gave almost identical results (data not shown), that is, adjustment for physical activity, smoking and hormone treatment made little difference.

The observed associations were of broadly equivalent magnitude and statistically significant for the relation between BMI, waist circumference, VAT, waist/height ratio or body fat percentage and all investigated indices of glucose metabolism. These obesity measures were positively associated with indices of glycaemia, and inversely associated with insulin sensitivity. One standard deviation difference in the obesity measures corresponded approximately to a 0.2 mmol/l higher fasting glucose, a 0.7 mmol/l higher 2 hour glucose, and 0.6–1.2 mmol/mol (0.06–0.1 per cent point) higher HbA1c. For the calculated indices of glucose metabolism the approximate differences per standard deviation increase in obesity measure were a 30% lower HOMA-S, a 20% lower Gutt’s index of insulin sensitivity, and a 100 unit higher Stumvoll’s index of beta-cell function.

The associations for SAT and VAT/SAT ratio largely showed the same direction as for the other obesity measures, but were less strong and not all statistically significant. Sex differences were more pronounced. For subcutaneous fat, all associations were stronger for women. For the VAT/SAT ratio, the associations were stronger for men. Among the statistically significant associations in Fig 4, sex differences were significant for the associations of SAT with HOMA-S (p = <.0001), Gutt’s index of insulin sensitivity (p = 0.01), and Stumvoll’s index of beta-cell function (p = 0.04). Correspondingly, sex differences were also significant for the associations of VAT/SAT ratio with HOMA-S (p = <.0001), Gutt’s index of insulin sensitivity (p = 0.001) and Stumvoll’s index (p = 0.002). The only other significant sex difference was found for the association of fat percentage and Stumvoll’s index (p = 0.03).

In a second analysis, the same model was repeated for ultrasound measures using non-standardised measures to allow clinical interpretation of the size of the beta-coefficients found. Further adjustment was made for i) waist circumference and ii) waist circumference and body fat. Table 2 shows selected results.

**Table 2:** Beta coefficients and 95% confidence intervals for associations of non-standardised measures of VAT, SAT and body fat percentage with indices of glucose metabolism.

		Only adjusted for confounders		Further adjustment for waist circumference		Further adjustment for waist circumference and body fat %	
Glucose markers	Obesity	Women	Men	Women	Men	Women	Men
FPG^1^	VAT	**0.09(0.06;0.11)**	**0.08(0.06;0.10)**	**0.05(0.01;0.08)**	**0.05(0.02;0.07)**	**0.05(0.01;0.08)**	**0.04(0.01;0.07)**
(mmol/l)	SAT	**0.08(0.03;0.13)**	0.05(-0.01;0.11)	-0.04(-0.10;0.02)	-0.03(-0.09;0.03)	**-0.08(-0.14;-0.01)**	-0.04(-0.10;0.02)
	Body fat %	**0.03(0.02;0.04)**	**0.04(0.03;0.04)**	0.01(0.00;0.03)	**0.03(0.01;0.04)**		
2hrPG^2^	VAT	**0.30(0.23;0.38)**	**0.28(0.22;0.33)**	**0.16(0.06;0.25)**	**0.19(0.12;0.27)**	**0.16(0.06;0.25)**	**0.17(0.09;0.25)**
(mmol/l)	SAT	**0.23(0.08;0.38)**	0.11(-0.07;0.29)	**-0.23(-0.40;-0.05)**	-0.17(-0.35;0.01)	**-0.28(-0.47;-0.08)**	**-0.20(-0.38;-0.02)**
	Body fat %	**0.09(0.07;0.12)**	**0.12(0.09;0.14)**	0.00(-0.04;0.05)	**0.08(0.03;0.12)** ^a^		
Hba1c (%)	VAT	**0.04(0.03;0.06)**	**0.04(0.03;0.05)**	**0.03(0.02;0.05)**	**0.04(0.02;0.05)**	**0.04(0.02;0.05)**	**0.03(0.02;0.05)**
	SAT	0.02(-0.01;0.04)	-0.03(-0.06;0.01)^**a**^	-0.04(-0.07;0.00)	**-0.06(-0.1;-0.03)**	-0.04(-0.07;0.00)	**-0.07(-0.10;-0.04)**
	Body fat %	0.01(0.00;0.01)	**0.01(0.01;0.02)**	0(-0.01;0.00)	0.01(0.00;0.02)^**a**^		
HOMA-S,	VAT	**-12.61(-14.47;-10.7)**	**-11.52(-12.96;-10.06)**	**-4.69(-7.17;-2.15)**	**-6.10(-8.08;-4.08)**	**-4.62(-7.04;-2.14)**	**-4.41(-6.42;-2.36)**
% change	SAT	**-19.48(-23.02;-15.77)**	**-10.55(-15.21;-5.64)** ^**a**^	-3.72(-8.38;1.18)	1.85(-3.11;7.06)	-0.65(-5.69;4.66)	4.33(-0.62;9.53)
	Body fat %	**-5.11(-5.74;-4.48)**	**-6.08(-6.70;-5.44)** ^**a**^	**-2.08(-3.29;-0.86)**	**-4.99(-6.07;-3.90)** ^**a**^		
ISI0_120,	VAT	**-8.15(-9.54;-6.75)**	**-7.70(-8.75;-6.63)**	**-3.96(-5.78;-2.11)**	**-5.03(-6.49;-3.55)**	**-3.95(-5.74;-2.12)**	**-4.03(-5.51;-2.53)**
% change	SAT	**-9.32(-12.16;-6.39)**	**-4.97(-8.50;-1.31)**	2.01(-1.61;5.77)	2.83(-0.84;6.64)	**4.18(0.27;8.24)**	**4.40(0.74;8.19)**
	Body fat %	**-2.90(-3.38;-2.43)**	**-3.69(-4.17;-3.22)** ^**a**^	**-0.96(-1.86;-0.05)**	**-3.12(-3.94;-2.30)** ^**a**^		
Stumvoll’s	VAT	**33.12(18.06;48.17)**	**36.66(25.24;48.09)**	14.57(-4.69;33.83)	10.10(-5.47;25.67)	14.12(-4.97;33.22)	2.30(-13.47;18.07)
index	SAT	**66.64(37.33;95.94)**	29.21(-5.58;64)	34.93(-0.77;70.63)	-17.33(-53.21;18.55)^**a**^	21.74(-16.54;60.03)	-27.04(-62.77;8.7)
	Body fat %	**13.72(8.87;18.56)**	**22.86(18;27.72** ^**a**^	**10.74(1.59;19.9)**	**20.41(11.99;28.82)**		

Significant associations are in bold.

^a^ indicates significant sex differences in the associations, (p<0.05).

^1^FPG: fasting plasma glucose.

^2^2hrPG: plasma glucose after 2 hours.

After adjustment for waist circumference, the associations for visceral fat remained significant for both sexes for all indices of glucose metabolism except Stumvoll’s index, and there were still no significant sex differences. Of note, the association with two hour glucose remained strong. For a given waist circumference, a 1 cm difference in visceral fat is associated with a 0.16 mmol/L higher 2 hour glucose level for women (p = 0.001) and a 0.19 mmol/L higher level for men (p<0.0001). These findings were not changed markedly with further adjustment for body fat percentage.

For subcutaneous fat, the associations were strongly attenuated or changed direction. For example, for two hour glucose, the associations change from being positive (and statistically significant for women) to a negative association, significant for women. Thus, for a given waist circumference a 1 cm difference in subcutaneous fat is associated with 0.23 mmol/l lower 2 hour level (p = 0.01) in women.

For body fat percentage several of the associations remained significant after adjustment for waist circumference. This marker of overall obesity was now significantly associated with Stumvoll’s index of betacell function whereas VAT and SAT were not. A 1% change in body fat is associated with a 11 unit higher Stumvoll’s index for women (p = 0.02) and 20 units higher for men (p<0.0001).

## Discussion

All the investigated measures of obesity were positively associated with indicators of glycaemia and inversely associated with indicators of insulin sensitivity. When evaluated separately, associations were of similar magnitude for visceral fat, BMI, waist circumference, waist-height ratio and body fat percentage. Associations for subcutaneous fat and the ratio of VAT/SAT were less strong. Even after adjustment for waist circumference, the association between the ultrasound measurements, primarily VAT, and indices of glucose metabolism remained statistically significant. For the associations between SAT and indices of glucose metabolism there was a trend toward a change of direction to a negative association upon adjustment for waist circumference.

Other studies have focused on the magnitude of the association between VAT and SAT, directly measured with CT or MRI, and metabolic risk. Their findings have added weight to the hypothesized role of VAT as a pathogenic fat depot of particular importance for metabolic health [7,25–28]. In particular, prospective studies have provided evidence that VAT is an independent predictor of incident pre-diabetes and diabetes [3–5]. Our results are consistent with previous work in finding an association between SAT and VAT and indices of glucose metabolism, and in finding a stronger association for VAT than for SAT—with the benefit that our study is based on a highly feasible one-dimensional assessment of VAT and SAT with ultrasonography rather than on methods requiring expensive equipment and offline interpretation of results, such as the two- or three-dimensional imaging with CT or MRI.

The Framingham Heart Study (FHS) examined cross-sectionally the association between abdominal fat distribution assessed by CT and various metabolic risk factors. In a large community-based primarily white population, both SAT and VAT were significantly associated with fasting plasma glucose and HOMA insulin resistance. The associations were stronger with VAT than with SAT. VAT but not SAT contributed significantly to risk factor variation after adjustment for BMI and waist circumference [7,25]. Similarly in our study, after adjustment for waist circumference and body fat percentage, associations between VAT and glucose outcomes are still significant for all outcomes except Stumvoll’s index of betacell function. And the strength of the associations between VAT and HOMA-S, ISI_0,120_ and 2hr glucose remain clinically relevant.

Some results in the FHS are directly comparable to ours. For example, after adjustment for waist circumference, one standard deviation increase in VAT is associated with 11.3% and 18.7% increase in HOMA insulin resistance for women and men respectively in our study (data not shown). In the FHS, using CT for assessing VAT and SAT, the same analysis gives ~13% (β = 0.12 for Log Homa-IR) [25]. Furthermore, a one standard deviation increase in VAT before adjustment for waist circumference is associated with an increase in fasting glucose in the FHS [7] of ~ 4.0 mg/dl = 0.2 mmol/l that is almost identical to that found in our study (Fig 4). Other studies also find similar results to ours. In a cross-sectional analysis, the association between abdominal fat distribution and glucose metabolism in the Health, Aging and Body Composition Study was investigated in a cohort of elderly participants [26]. The study found a standardized beta-coefficient for the significant association of VAT assessed by CT and fasting plasma-insulin of ~ 0.35. In our study the corresponding significant value is 0.3 (data not shown).

The associations we find between SAT and glucose outcomes tend to change direction after adjustment for waist circumference. In particular, the beta-coefficient for the association with 2hr glucose changes from 0.23 mmol/l before adjustment to—0.23mmol/l after adjustment. In line with previous research, this adds weight to the theory of SAT as a protective metabolic sink. Based on the strength of the associations for the VAT/SAT ratio and glucose outcomes in our study (Fig 4), we would argue that VAT and SAT are best analysed separately, with the possibility of adjusting one for the other.

Our study includes a 3 point OGTT, enabling us to examine the association between abdominal fat distribution and Gutt’s index of insulin sensitivity (ISI_0,120_), 2hr glucose, and Stumvoll’s index, i.e. indices of glucose metabolism other than those derived from fasting values of glucose and insulin. While HOMA-S can be seen as a marker of hepatic insulin sensitivity, ISI_0,120_ adds information on peripheral insulin sensitivity. The associations found for ISI_0,120_ and 2hr glucose are similar to results of the same analysis done in another study of over 3000 Inuit, where abdominal fat distribution has been assessed with the same ultrasound method [13].

Interestingly, we found that body fat but neither VAT nor SAT were significantly associated with Stumvoll’s index of beta cell function after adjustment for waist circumference. No other study has reported results from this type of analysis. This suggests that the effect of VAT on glucose metabolism is via insulin sensitivity not insulin secretion. Body fat percentage, on the other hand, may have an association with insulin secretion.

Few sex differences are found in our non-standardised analyses (Table 2). For the association between SAT and HOMA-S, there is a significantly stronger association for women. This disappears after adjustment for waist circumference, indicating that SAT in women is perhaps a marker of overall adiposity in our study. Several studies, including the FHS, whose participants were in a similar BMI range to ours, found a stronger association for women between VAT and metabolic risk factors including fasting plasma glucose [4,7]. On the other hand, the Dallas Heart Study of obese participants found that the associations of VAT and SAT with metabolic phenotypes are similar in men and women [27].

This study extends previous work by finding consistent results using more accessible ultrasound technology instead of CT/MRI. We also extend current literature by examining a population with a different, higher risk of diabetes profile than that of a general population.

A major strength of this study is the large number of participants with fat distribution measured by imaging techniques. In epidemiology, waist circumference is typically used to quantify central obesity. Our findings strengthen the case for the use of ultrasonography to achieve a more detailed ascertainment of fat distribution in future epidemiological studies. We obtained the most precise measures of glucose homeostasis achievable in an epidemiological setting. Finally, we have information on relevant confounders such as physical activity.

A limitation of our analysis is its cross-sectional design. However, our results are consistent with those found by prospective studies. The majority of our study participants are Caucasians aged in their 60’s with weight in the normal/overweight range. We cannot generalise our results to other populations.

In this population of people at higher risk of diabetes than a general population, VAT and SAT assessed by ultrasonography are significantly associated with indices of glucose metabolism. Even after adjustment for waist circumference and body fat percentage primarily VAT adds clinically relevant information regarding risk of diabetes. Ultrasonography should be considered as a means of assessing abdominal fat distribution in epidemiological studies where CT/MRI is not accessible.

## Supporting Information

S1 Dataset The dataset for this study. (SAS7BDAT)
